# Highly sensitive and stable zwitterionic poly(sulfobetaine-3,4-ethylenedioxythiophene) (PSBEDOT) glucose biosensor†
†Electronic supplementary information (ESI) available. See DOI: 10.1039/c7sc05104b


**DOI:** 10.1039/c7sc05104b

**Published:** 2018-01-24

**Authors:** Haiyan Wu, Chen-Jung Lee, Huifeng Wang, Yang Hu, Megan Young, Yu Han, Fu-Jian Xu, Hongbo Cong, Gang Cheng

**Affiliations:** a Department of Chemical and Biomolecular Engineering , University of Akron , Akron , Ohio 44325 , USA . Email: hcong@uakron.edu ; http://www.uakron.edu/engineering/about-us/people-directory/bio-detail.dot?u=hcong; b Department of Chemical Engineering , University of Illinois at Chicago , Chicago , Illinois 60607 , USA . Email: gancheng@uic.edu ; https://che.uic.edu/k-teacher/gang-cheng-ph-d/; c Department of Mechanical Engineering , University of Akron , Akron , Ohio 44325 , USA; d Key Laboratory of Carbon Fiber and Functional Polymers (Ministry of Education) , Beijing Laboratory of Biomedical Materials , Beijing University of Chemical Technology , Beijing 100029 , China

## Abstract

A zwitterionic poly(sulfobetaine-3,4-ethylenedioxythiophene) (PSBEDOT)-based glucose biosensor was fabricated *via* encapsulating glucose oxidase (GOx) in a one-step electropolymerization method. Integrating conductivity and hydrophilic properties, PSBEDOT provides a great framework for GOx immobilization and stabilization. The anti-fouling, high-sensitivity, and long-term stability properties of PSBEDOT–GOx make it a promising platform for long-term and continuous glucose monitoring.

## Abstract 2

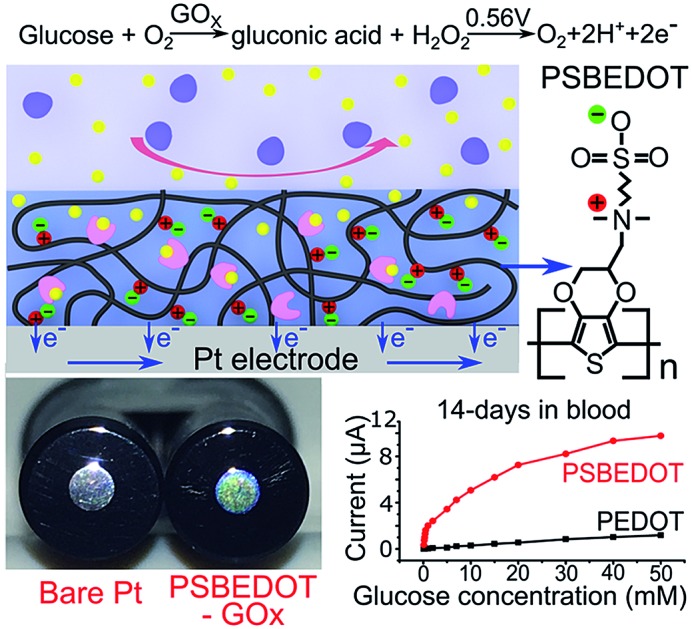
A zwitterionic poly(sulfobetaine-3,4-ethylenedioxythiophene) (PSBEDOT)-based glucose biosensor was fabricated *via* encapsulating glucose oxidase (GOx) in a one-step electropolymerization method.

## 

Determination of glucose concentration is important in many fields, including the food industry,^1^,^2^ environmental monitoring,^2^ the biotech industry, and clinical applications.^3^,^4^ It is especially critical in diabetes, which is one of the leading causes of death and disability in the world. Due to the sustained prevalence of diabetes, significant efforts have been devoted to developing the easily processed, stable, low-cost, and accurate glucose biosensors. Electrochemical enzymatic biosensors, especially based on the amperometric method,^5^,^6^ are commonly used for glucose detection due to their high sensitivity and selectivity. Numerous electrochemical enzymatic biosensors have been made by directly immobilizing oxidoreductases and mediators on several types of electrode surface; however, these devices usually suffer from an unstable response due to poor electron transfer between the electrode and the enzyme,^5^ and and rapid loss of enzyme activity.^6^ Recently, conducting polymers have been utilized as the enzyme-immobilization matrixes since they provide a three-dimensional electrically conducting network, and thus lead to rapid electron transfer to the electrode.^7^ While there are several ways of immobilizing enzymes in a conducting polymer matrix, entrapping the enzymes simultaneously during electropolymerization of the monomer in a single-step process is one appears more attractive due to its simplicity and its ability to control film thickness and enzyme loading.

Several types of conducting polymers, including polyaniline (PANi),^8^–^11^ polypyrrole (PPy),^12^–^14^ and polythiophene,^15^,^16^ have been deposited electrochemically with glucose oxidase (GOx) onto metal electrodes to construct glucose biosensor probes. Among the conducting polymers, poly(3,4-ethylenedioxythiophene) (PEDOT) has attracted significant attention since it offers high stability,^17^ the ability to be electropolymerized with a low oxidation potential^18^ in aqueous media,^19^ and excellent conductivity.^20^ Nien *et al.* reported an amperometric glucose biosensor that entraps GOx in a PEDOT film in a single electropolymerization step.^21^ With ferrocene as a mediator, the current retains 80% of its original value after 18 days, although no storage conditions were provided in the paper. Invernale *et al.* also fabricated a microneedle electrode for glucose sensing using the same single-step electropolymerization method,^22^ which shows the potential for painless sampling. However, this only showed good stability with 95% activity within 7 days when stored either in air or in phosphate-buffered saline (PBS). The most common and challenging problems for current enzymatic glucose biosensors are the insufficient long-term stability attributed to the intrinsic nature of the enzyme and relatively low sensitivity in complex biological fluids, such as human blood, due to the non-specific protein adsorption and cell attachment, originating from the hydrophobic or charged surface of conventional conducting polymers.

A continuous glucose sensor with long stability, high specificity, and high selectivity, which can provide reliable monitoring under complex conditions, is urgently needed for diabetic management. Recently, many studies have demonstrated that zwitterionic materials can effectively resist cell attachment and protein adsorption, and thus are suitable for complex media applications,^23^–^26^ such as serving as a biosensor for a blood glucose monitor.^27^–^30^ Yang *et al.* reported that zwitterionic poly(carboxybetaine methacrylate) (PCBMA) hydrogels on glucose sensor surfaces show an ultra-low fouling background to maintain a highly stable response, even in complex medium, human blood serum.^31^ In addition, PCBMA can improve stability with the retention of binding affinity or bioactivity of PCBMA–trypsin conjugate.^32^ Although the PCBMA-based glucose sensor shows great improvement as a blood glucose monitor, it is still limited by several issues: (1) most current zwitterionic materials only offer good biocompatibility for the biosensor, but compromise the electrochemical properties of the conductive substrate due to the non-conductive polymer backbone;^33^–^35^ (2) the fabrication of the biosensor is complicated and time consuming since the conductive component generally needs to mix or covalently bind with the biocompatible but non-conductive moieties. To address the challenges of non-conductive zwitterionic polymers, Cao *et al.* developed two zwitterionic conjugated polymers: poly(sulfobetaine-3,4-ethylenedioxythiophene) (PSBEDOT) and poly(carboxybetaine thiophene) (PCBTh). PSBEDOT and PCBTh can conduct ions, provide excellent anti-fouling properties *via* zwitterionic side chains, and conduct electrons *via* conjugated backbones.^36^,^37^ Both polymers show great potential for electrochemical biosensing.

We hypothesized that zwitterionic conjugated polymers can provide a more suitable environment to stabilize the enzyme due to their hydrophilicity and hydration property, and thus prevent biofouling. Herein, as shown in Scheme 1, we utilized PSBEDOT, which integrates both the conducting and anti-fouling properties,^36^ as a matrix for entrapping GOx onto a platinum electrode in a single electropolymerization step. The PSBEDOT–GOx electrode was expected to provide several desired properties for an enzyme-based electrochemical biosensor, including long-term stability, high selectivity and sensitivity, short response time, accurate measurement, stable response in complex media due to superior anti-fouling properties, and enhanced electrical conductivity within one component. The surface morphology and roughness, protein adsorption, response time, chronoamperometry properties, and stability in dry conditions, PBS, and human blood plasma of the PSBEDOT glucose biosensor were systematically investigated in this work.

**Scheme 1:**
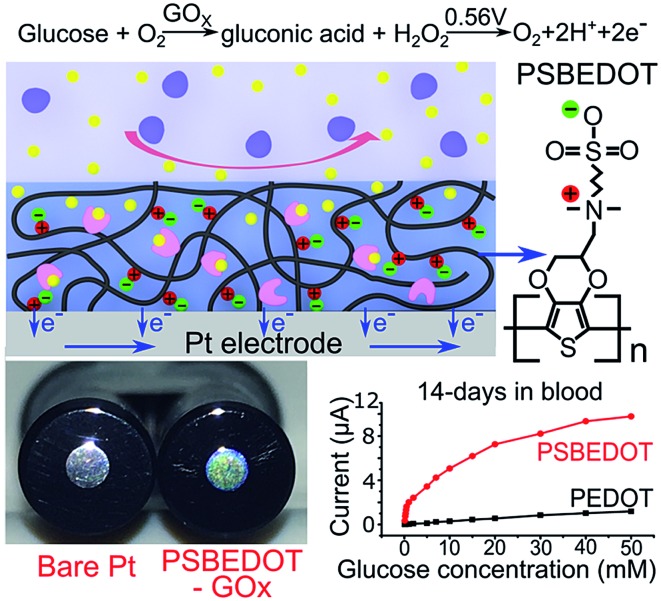
Zwitterionic poly(sulfobetaine-3,4-ethylenedioxythiophene) (PSBEDOT) glucose biosensor.

The PSBEDOT–GOx electrode was conveniently synthesized *via* a one-pot electropolymerization process. SBEDOT monomers were electropolymerized at the interface of a Pt electrode, and the SBEDOT solution also contains 1 mg mL^–1^ GOx. GOx was encapsulated in the PSBEDOT network, when PSBEDOT was deposited on the Pt electrode surface. After 30 s of polymerization, a uniform, dark-blue film of PSBEDOT–GOx was formed on the Pt electrode.

Electrodeposition of highly water-soluble monomers on conductive or semi-conductive substrates has been a challenging issue. First, water-soluble monomers are very limited. Second, the polymers or oligomers produced cannot deposit on the surfaces easily due to their high solubility in the aqueous phase. The unique properties of PSBEDOT offer both the high solubility of the monomer in water and the ease of deposition of the polymer. The zwitterionic sulfobetaine side chain of sulfobetaine-3,4-ethylenedioxythiophene (SBEDOT) significantly increases the solubility of the monomer, and electrostatic interactions among sulfobetaine sidechains of PSBEDOT lead to the deposition of the polymer on the electrodes. Although the unmodified poly(3,4-ethylenedioxythiophene) (PEDOT) has been used for numerous biomedical applications because of the water solubility of 3,4-ethylenedioxythiophene (EDOT) monomer and the hydrophilicity of PEDOT, the solubility of EDOT in water is moderate. At a concentration of 80 mM, it will take over 12 hours to partially dissolve EDOT. These properties of SBEDOT allow people to use environmentally-friendly aqueous-based processes and provide the freedom to further optimize the sensors.

PSBEDOT functions as a three-dimensional conductive polymer matrix for GOx encapsulation, as well as an electrical mediator for transducing the signal produced *via* a redox reaction of glucose. In any polymeric GOx-based glucose biosensor, glucose needs to diffuse through the polymer matrix and bind to GOx, which converts the glucose to d-glucono-1,5-lactone and produces hydrogen peroxide. In PSBEDOT–GOx sensor, hydrogen peroxide was further oxidized at the platinum surface at +0.56 V potential relative to Ag/AgCl/saturated KCl. A current due to the oxidation of hydrogen peroxide was detected and can be related to the concentration of glucose. In this process, the retention of GOx bioactivity plays a key role in the behavior of the enzyme-based glucose sensor. It has been reported that the activity of the immobilized enzyme depends upon the surface area, porosity, roughness, hydrophilic property of the conducting polymer matrix, and electropolymerization conditions.^2^ Hence, the immobilization of the enzyme, surface morphology, and roughness of the PSBEDOT–GOx electrode were characterized using a fluorescence microscope, scanning electron microscope (SEM), and surface profiler in our study.

The PEDOT–GOx electrode was prepared under the same electropolymerization conditions as a reference. To confirm the encapsulation of the enzyme, GOx was labeled with fluorescein isothiocyanate (FITC) as a tracer. As shown in Fig. s1(a),† bare PSBEDOT films showed a very low fluorescence signal under the green fluorescent protein (GFP) channel. The PSBEDOT–GOx surface showed a strong fluorescent signal, indicating the immobilization of FITC-labeled GOx on the surface of the PSBEDOT electrode (Fig. s1(b)†). The surface morphology and roughness of the PSBEDOT–GOx electrode surface were then characterized using SEM and a surface profiler, respectively. After GOx was encapsulated by PSBEDOT, the topography of the surface was changed considerably, and the PSBEDOT–GOx surfaces showed quite smooth morphology due to the loading of GOx (Fig. s2(a, b)†). SEM results were consistent with the fluorescence microscope results. From Table s1,† the surface roughness measurement also indicated a smoother and thinner surface after GOx encapsulation. The root mean square of surface roughness (*R*_q_) decreased from 353.21 ± 17.07 nm (PSBEDOT) to 306.19 ± 38.92 nm (PSBEDOT–GOx) and the thickness decreased from 1.36 ± 0.06 μm (PSBEDOT) to 0.74 ± 0.07 μm (PSBEDOT–GOx). As shown in Fig. s2(b),† the PSBEDOT–GOx surfaces are rougher and thinner (*R*_q_ = 306.19 ± 38.92 nm, *T* = 0.74 ± 0.07 μm from Table s1†) when compared to the PEDOT–GOx surfaces (Fig. 2(d); *R*_q_ = 255.79 ± 18.99 nm, *T* = 0.94 ± 0.09 μm from Table s1†). The rougher surface provides a higher surface area to load GOx. The thinner surface reduces the distance for better mass transport for glucose to diffuse to GOx and for hydrogen peroxide to reach Pt surface,^2^ thus increasing the sensor sensitivity and reducing response time.^38^ In addition, the pores on the PSBEDOT–GOx film were more compact than on the PEDOT–GOx surface, which may have reduced the leaching of the enzymes from the surface.

**Fig. 1:**
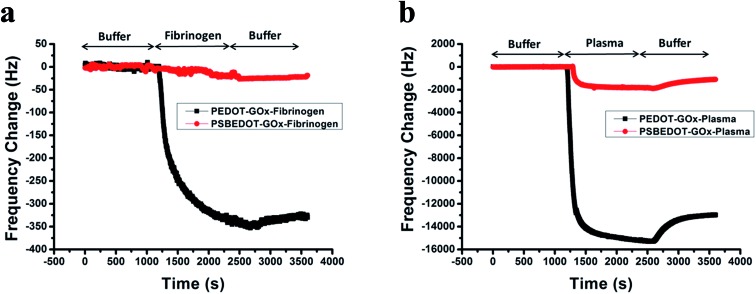
eQCM frequency shift of fibrinogen (a) and human blood plasma adsorption (b) on the PSBEDOT–GOx (red) and PEDOT–GOx (black) surfaces.

**Fig. 2:**
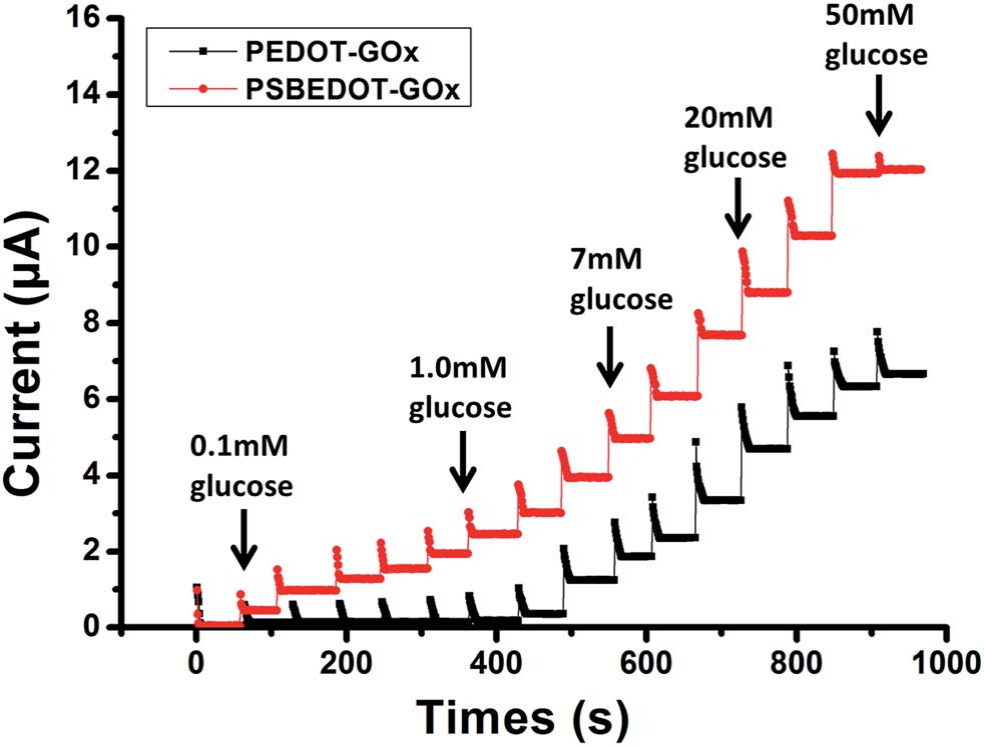
Amperometric response of the PSBEDOT–GOx (red) and PEDOT–GOx (black) biosensors to the successive addition of glucose in PBS; the concentration of glucose was increased from 0.1 mM to 50 mM.

Non-specific protein adsorption remains a major barrier to the development of a stable and sensitive glucose biosensor, especially for continuous glucose monitoring, since non-specific protein adsorption increases the noise-to-signal ratio, leads to enzyme-activity loss, and blocks the electrode surface.^26^,^39^,^40^ In this study, the amount of protein adsorbed on the PSBEDOT–GOx surface was quantified using an electrochemical quartz crystal microbalance (eQCM). The protein adsorption of the PEDOT–GOx surface was also evaluated. During the protein adsorption experiments, the frequency change of the oscillating quartz crystal was monitored over time. As shown in Fig. 1(a), for fibrinogen adsorption, the frequency shifted ∼25 Hz for the PSBEDOT–GOx surface after the introduction of protein solution and PBS that washed away loosely adsorbed protein. Fibrinogen adsorption on the PSBEDOT–GOx surface was 7.5% compared with that on the PEDOT–GOx surface (Δ*f* = 335 Hz). For 100% human blood plasma adsorption (Fig. 1(b)), the frequency shift increased to 1100 Hz for the PSBEDOT–GOx surface, which is 8.4% compared with that of the PEDOT–GOx surface (Δ*f* = 13 000 Hz). These results indicate that GOx encapsulation did not compromise the anti-fouling property of the PSBEDOT surface,^36^ and it still showed excellent protein resistance to both fibrinogen and 100% blood plasma compared to the PEDOT–GOx surface. Our results suggest that the PSBEDOT–GOx electrode has significant advantages for complex media or *in vivo* applications because of its anti-fouling properties.

The amperometric response of the PSBEDOT–GOx glucose biosensor to the successive addition of glucose (from 0.1 mM to 50 mM) with a sampling time of 60 s at each concentration was measured (Fig. 2). A PEDOT–GOx glucose biosensor was also tested under the same conditions. Three electrodes were prepared and studied for each type of glucose biosensor, and the sensors showed good repeatability and consistency. The response time, which is defined as the sensing current reaching 95% of the steady-state current (disturbance ≤ 20 nA), was 4–8 s for PSBEDOT–GOx the glucose sensor and 8–12 s for PEDOT–GOx glucose sensor. The steady current was recorded for each glucose concentration, and the curve for the relationship between current response and glucose concentration is plotted in Fig. 3. It was observed that both PSBEDOT–GOx and PEDOT–GOx sensors showed a good linear relationship (*R*^2^ > 0.97) between the current and glucose concentration at an intermediate glucose concentration ranging from 1 mM to 20 mM, and both curves became non-linear asymptotic at higher glucose concentrations (>20 mM, data not shown). However, only the PSBEDOT–GOx sensor displayed better linearity (*R*^2^ = 0.9874) at the lower glucose concentration range from 0.1 mM to 0.5 mM, whereas PEDOT–GOx did not respond noticeably to glucose. The average sensitivity calculated for the PSBEDOT–GOx and PEDOT–GOx sensors were 12.63 μA cm^–2^ mM^–1^ and 7.54 μA cm^–2^ mM^–1^, respectively, at the glucose range of 1 mM to 20 mM. These results indicate that PSBEDOT–GOx has higher sensitivity than the reported PEDOT–GOx glucose sensor.^15^,^21^,^41^ It is worth mentioning that the enzyme concentration for PSBEDOT–GOx in electropolymerization solution was 1 mg mL^–1^ in our work, which was much less than for other glucose biosensors (10 mg mL^–1^) reported previously.^22^,^41^,^42^ In other words, the PSBEDOT glucose biosensor was more economical in achieving a higher sensitivity. The average sensitivity for the PSBEDOT–GOx and PEDOT–GOx sensor at the lower glucose concentration range (0.1 mM to 0.5 mM) was 110.64 μA cm^–2^ mM^–1^ and 2.63 μA cm^–2^ mM^–1^, respectively. The sensitivity of the PSBEDOT glucose sensor at low glucose concentrations (<0.5 mM) is much higher than other reported glucose sensors, including PANi-based hydrogel,^34^ high-surface-area Pt-polypyrrole,^38^ and carbon nanotubes.^43^ The exceptionally high sensitivity contributed to the excellent anti-fouling properties and high roughness of the PSBEDOT–GOx surface, resulting in high enzyme activity, improved H_2_O_2_ electrooxidation efficiency,^38^ and better mass transfer.

**Fig. 3:**
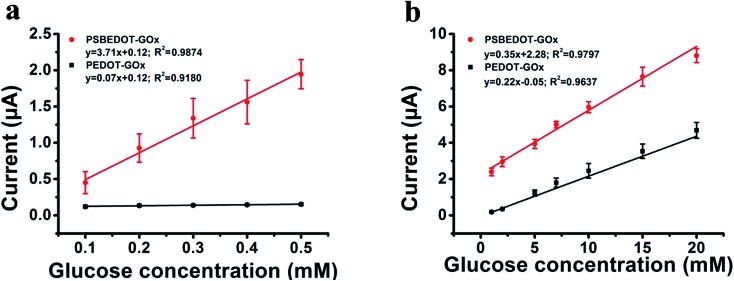
Corresponding calibration plot showing amperometric response *vs.* glucose concentration: (a) 0.1 mM to 0.5 mM; (b) 1 mM to 20 mM. Replicates = 3.

The most challenging issue for enzyme-based biosensors is their limited long-term stability, especially for continuous glucose monitoring due to the instability of the enzyme. Hence, the long-term stability of the PSBEDOT–GOx and PEDOT–GOx glucose biosensors was investigated under both dry (in an empty vial) and wet (PBS) conditions at room temperature. The performance of the PSBEDOT–GOx glucose sensor was not significantly affected by any of these storage conditions, and the current signal was kept at almost 100% of original current in either dry or wet conditions after 21 days of storage, which is much longer than most conducting polymer-based enzymatic glucose biosensors reported in the literature.^21^,^44^ However, only <38% and <35% signal remained for the PEDOT–GOx sensor under dry (Fig. 4(b)) and wet (Fig. 4(d)) conditions, respectively. The signal of the PSBEDOT–GOx sensor disappeared after 77 days under dry conditions and after 70 days for the wet conditions, while the signal of the PEDOT–GOx sensor diminished to 0 after 30 days and 49 days. These results show that the PSBEDOT glucose sensor has much better stability than the PEDOT glucose sensor under both dry and wet conditions. The exceptionally long-term stability could be attributed to (1) the compact morphology and hydrophilicity characteristics of the PSBEDOT surface provides a much better matrix to reduce enzyme-activity loss and leaching, which are the main causes of the sensor failure;^2^,^45^ (2) the ultra-low fouling property of the PSBEDOT–GOx surface reduces the electrode fouling (often called electrode passivation) that causes sensor deactivation;^45^ (3) the zwitterionic polymer has been shown to increase the protein stability.^32^

**Fig. 4:**
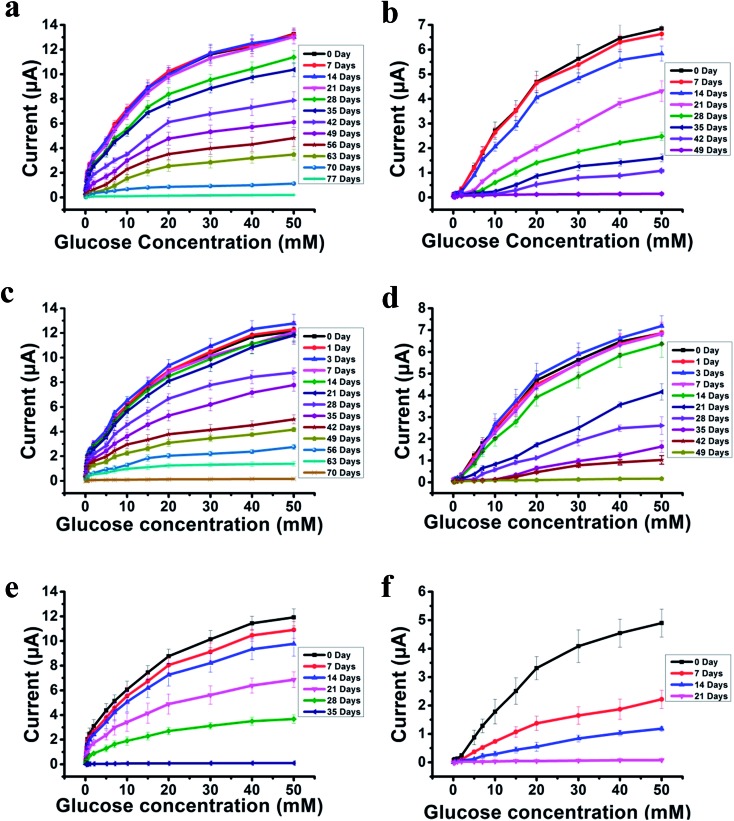
Response of PSBEDOT–GOx (a) and PEDOT–GOx (b) sensors to glucose from 0.1 mM to 50 mM after long-term storage under dry conditions. The response of PSBEDOT–GOx (c) and PEDOT–GOx (d) sensors to glucose from 0.1 mM to 50 mM after long-term storage under wet conditions. Current response of PSBEDOT–GOx (e) and PEDOT–GOx (f) glucose sensors incubated in 100% human blood plasma at 0, 7, 14, 21, 28 and 35 days until the current signal was lost completely as a function of glucose concentration (0.1–50 mM). Replicates = 3.

One goal of the glucose biosensor is for continuous real-time *in vivo* monitoring that can provide maximal information for patients with diabetes to make fast and optimal therapeutic interventions (*i.e.*, insulin delivery). We first investigated the performance of the PSBEDOT–GOx sensor in 100% human blood plasma. After the initial blood contact, the average sensitivity was 10.54 μA cm^–2^ mM^–1^ and 5.33 μA cm^–2^ mM^–1^ for PSBEDOT and PEDOT sensors, respectively, at a glucose concentration range between 1 mM to 20 mM. After the initial contact, the sensitivity of PSBEDOT–GOx and PEDOT–GOx decreased 16.5% and 29.3%, respectively, compared to PBS. However, the sensitivity for the PSBEDOT–GOx sensor remain exceptionally high at the low glucose concentration range between 0.1 mM to 0.5 mM in human blood plasma, which was 124.57 μA cm^–2^ mM^–1^. Meanwhile, both the sensitivity and the linearity (as shown in Fig. 4(e)) of the PSBEDOT glucose sensor did not decline significantly (within 10%) after being stored in human blood plasma over 14 days, which was even better than the reported zwitterionic PCBMA hydrogel-based glucose sensor.^34^ However, for the PEDOT–GOx sensor, more than 50% of the signal was lost after only 7 days (Fig. 4(f)). In human blood plasma, more than 50% of the signal remained for PSBEDOT–GOx after 3 weeks; however, the signal of the PEDOT–GOx sensor was completely lost after 3 weeks. The excellent sensitivity and long-term stability of the PSBEDOT sensor was also attributed to the ultra-low fouling property of the PSBEDOT surface that decreased the loss of enzyme from the surface, and reduced the surface deactivation by fouling. We believe the performance of PSBEDOT–GOx can be further enhanced by improving the PSBEDOT anti-fouling properties and reducing the leaching of the enzymes. Our results indicate that PSBEDOT with both zwitterionic properties and conductivity can improve the enzyme stability/activity for biosensing applications.^32^

In conclusion, a zwitterionic PSBEDOT–GOx glucose sensor was fabricated in a facile single-step procedure by encapsulating GOx into a PSBEDOT network during the electropolymerization process. The PSBEDOT–GOx sensor demonstrated high sensitivity for low glucose concentration measurement and a much better stability for both dry and wet conditions compared to the PEDOT–GOx glucose sensor. The significantly improved stability and sensitivity of the PSBEDOT–GOx sensor is due to the strong hydration of the zwitterionic side chains that stabilizes the enzyme, the rougher surface for GOx immobilization, and ultra-low fouling properties to minimize the contact of the biological molecules with the electrode surface. PSBEDOT–GOx shows much higher stability in 100% human blood plasma, and the current signal remains over 90% after being stored in human blood plasma for 14 days. In this study, PSBEDOT demonstrates great potential for electrochemical biosensing applications since it may significantly increase the performance and service life, minimize foreign body reaction, and improve biocompatibility.

## Conflicts of interest

There are no conflicts to declare.

## Supplementary Material

Supplementary information
