# Supplementary material for: Highly sensitive and stable zwitterionic poly(sulfobetaine-3,4-ethylenedioxythiophene) (PSBEDOT) glucose biosensor
Source: Chem Sci. 2018 Jan 24;9(9):2540–6. doi: 10.1039/c7sc05104b (PMC5911821; doi:10.1039/c7sc05104b)
Supplement: Supplementary file 1 [file SC-009-C7SC05104B-s001.pdf]

Electronic Supplementary Material (ESI) for Chemical Science.
This journal is © The Royal Society of Chemistry 2018

Supporting Information:

Highly sensitive and stable zwitterionic poly(sulfobetaine 3,4-ethylenedioxythiophene) (PSBEDOT) glucose biosensor

Haiyan Wu,[a] Chen-Jung Lee,[a] Huifeng Wang,[b] Yang Hu,[b] Megan Young,[b] Yu Han,[c] Fu-Jian Xu,[d] Hongbo Cong*[a] and Gang Cheng*[b]

[a] Department of Chemical and Biomolecular Engineering, University of Akron, Akron, Ohio 44325, USA

[b] Department of Chemical Engineering, University of Illinois at Chicago, Chicago, Illinois 60607, USA

[c] Key Laboratory of Carbon Fiber and Functional Polymers (Ministry of Education), Beijing Laboratory of Biomedical Materials, Beijing University of Chemical Technology, Beijing 100029, China

Corresponding Author

* Dr. Gang Cheng E-mail: gancheng@uic.edu

Department of Chemical Engineering, University of Illinois at Chicago, Chicago, Illinois 60607, USA

* Dr. Hongbo Cong E-mail: hcong@uakron.edu

Department of Chemical and Biomolecular Engineering, University of Akron, Akron, Ohio 44325, USA

Table of Contents

1. Chemicals and general instruments S2

2. PSBEDOT-GOx sensor electrode preparation S3

3. Fluorescence microscope analysis, scanning electron microscope and surface profiler S3

4. Resistance to protein adsorption S4

5. Chronoamperometry and stability test S5

Experimental methods

1. Chemicals and general instruments

Glucose oxidase (GOx) type II from *Aspergillus niger*, 3,4-Ethylenedioxythiophene, lithium perchlorate, phosphate buffer saline (PBS, pH 7.4), platinum wire counter electrode and Indium tin oxide (ITO) coated PET were purchased from Sigma Aldrich (St. Louis, MO, USA). D(+)-Glucose Monohydrate, human fibrinogen, human blood plasma and NHS-Fluorescein were purchased from EMD Chemicals Inc (Gibbstown, NJ, USA), Calbiochem (San Diego, CA, USA), BioChemed Services (Winchester, VA, USA) and Thermo Fisher Scientific (Waltham, MA, USA), respectively. DI water used throughout the experiments was purified using a Millipore Milli-Q Direct 8 Ultrapure Water system (Billerica, MA, USA). 10 MHz Au coated quartz crystal was purchased from Gamry Instruments Inc (Warminster, PA, USA). 2 mm diameter platinum working electrode and Ag/AgCl saturated in KCl reference electrode was purchased from CH Instruments (Austin, TX, USA) and Gamry Instruments Inc (Warminster, PA, USA). All electrochemical experiments, including galvanostatic electro-polymerization and amperometric measurements, were performed with a Gamry Reference 600 potentiostat (Warminster, PA, USA). Protein adsorption test for various electrode surfaces was performed with a Gamry electrochemical quartz crystal microbalance (Warminster, PA, USA). The surface morphology and roughness of all the samples was observed with TESCAN LYRA3 field emission scanning electron microscope (FE-SEM) (Warrendale, PA, USA) and Bruker DektakXT surface profiler (Billerica, MA, USA).

2. PSBEDOT-GOx sensor electrode preparation

Sulfobetaine-3.4-ethylenedioxythiophene (SBEDOT) was firstly synthesized as we previously reported.[1] A platinum disc electrode with diameter of 2 mm was polished by micropolish powders (0.05 μm particle size) and then washed with DI water. The electrode was then washed through sonicating in DI water for 5 min and dried with air. Fresh electro-polymerization solution contained 80 mM SBEDOT and 100 mM $LiClO_4$. Then, 5 mg of GOx was added to 5 mL of electro-polymerization solution. To achieve the highest sensor sensitivity without compromising the morphology of the surface, 1 mg/mL of glucose oxidase was chosen for the PSBEDOT-GOx sensor electrode preparation. The enzyme sensor electrode was prepared by incorporating GOx simultaneously onto the platinum electrode through PSBEDOT electro-polymerization process in a three-electrode electrochemical system. An Ag/AgCl/saturated in KCl electrode and a platinum wire were used as reference electrode and counter electrode, respectively. Electro-polymerization was carried out through galvanostatic method with 1.59 mA/cm^2 for 30s. PEDOT-GOx electrode was also prepared through the same procedure as a reference.

3. Fluorescence microscope analysis, scanning electron microscope and surface profiler

To characterize the glucose oxidase encapsulation, glucose oxidase that labeled with NHS-fluorescein before incorporated into PSBEDOT matrix during electro-polymerization process, and PSBEDOT with and without GOx surfaces were imaged with an Olympus IX81 fluorescence microscope under a 20X objective lens through GFP filter (494/518nm).

To characterize the surface morphology of PSBEDOT with or without GOx surfaces, two samples of PSBEDOT and PSBEDOT-GOx were prepared on ITO substrates through the same method as described before for scanning electron microscope (SEM) analysis. Fine gold films were sputtered on the samples for higher conductivity and better observation. Superficial observations were performed on all the species with different magnitudes. Surface roughness of all the samples was directly measured by a surface profiler.

4. Resistance to protein adsorption

The adsorbed amount of 1 mg/mL of human fibrinogen and 100% human blood plasma on the PSBEDOT-GOx and PEDOT-GOx surfaces was quantified by Gamry eQCM. The mass changes were measured by monitoring the resonant frequencies of an oscillating quartz crystal. PSBEDOT-GOx and PEDOT-GOx surfaces were electropolymerized on the 10MHz Au coated quartz crystal using the same method as described above. In a typical procedure, the PSBEDOT-GOx coated oscillating quartz crystal was first exposed to PBS until a stable baseline was established. Then, 1 mg/mL of fibrinogen solution was flowed through for 20 min and then changed to PBS for 20 min to wash away the weakly adsorbed fibrinogen on the surface. The change of frequency of the oscillating quartz crystal was monitored, which indicated the amount of protein adsorbed. Same procedure was operated for the 100% human blood plasma adsorption test. The protein adsorption on the PEDOT-GOx surface was measured with the same procedure. All the experiments were repeated twice to guarantee the accuracy.

5. Chronoamperometry and stability

Chronoamperometry was used to measure the current change of PSBEDOT glucose biosensor due to the glucose additions. A 1.0 M glucose solution in PBS (pH 5.5) was prepared and added sequentially into sensing cell for desired concentration. A magnetic stir bar was used to ensure good mixing within the cell. The prepared PSBEDOT-GOx Pt electrode, Ag/AgCl saturated in KCl, and a platinum wire were used as working electrode, reference electrode, and counter electrode, and all electrodes were submersed in glucose solution together to form the cell. The current data was collected at each glucose concentration point (0.1 mM ~ 50 mM) by potential static method at 0.56 V when the current response achieved a steady station (current disturbance less than ± 20 nA). Therefore, the relationship curve for the current response and glucose concentration can be built. For each test, three PSBEDOT-GOx electrodes were used.

For long-term stability test, the electrode was stored in three different conductions: in air, PBS with 0.1 mg/mL sodium azide and human blood plasma at 4 ℃ before the next test for a selected time interval. The same three electrodes system as previous chronoamperometry test was used, and the current data for different glucose concentrations was also collected by applying 0.56 V when obtained a steady state. All experiments were operated at room temperature, and the solutions were stirred continuously during the experiment. The current response of glucose for the PSBEDOT-GOx was continuously recorded until no current response was detected. The curves for the relationship between current response and glucose concentration were built for each storage period. PEDOT-GOx electrode was also tested under the same method for comparison. For each test, three PSBEDOT-GOx electrodes were used.

Surface	Bare ITO	PSBEDOT	PEDOT	PSBEDOT-GOx	PEDOT-GOx
Rq (nm)	5.3±2.5	353.2±17.1	451.7±43.9	306.2±38.9	255.8±19.0
Thickness (μm)	/	1.36±0.06	1.28±0.23	0.74±0.07	0.94±0.09

Table s1**.** Surface roughness (Rq) and thickness (T) of polymer-GOx surfaces on the ITO surface through galvanostatic method with 1.59 mA/cm^2 for 30 s.

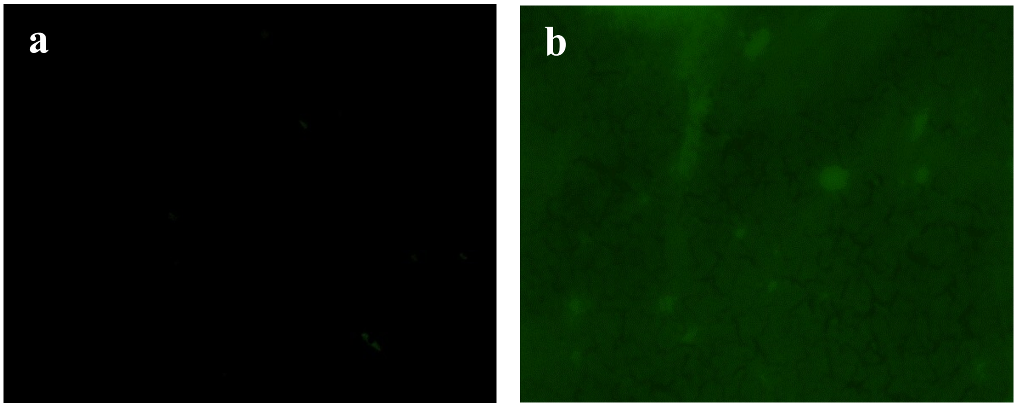

Figure s1. Fluorescence microscope images of (a) PSBEDOT and (b) PSBEDOT-GOx surfaces electro-polymerized on the ITO surface through galvanostatic method with 1.59 mA/cm^2 for 30 s.

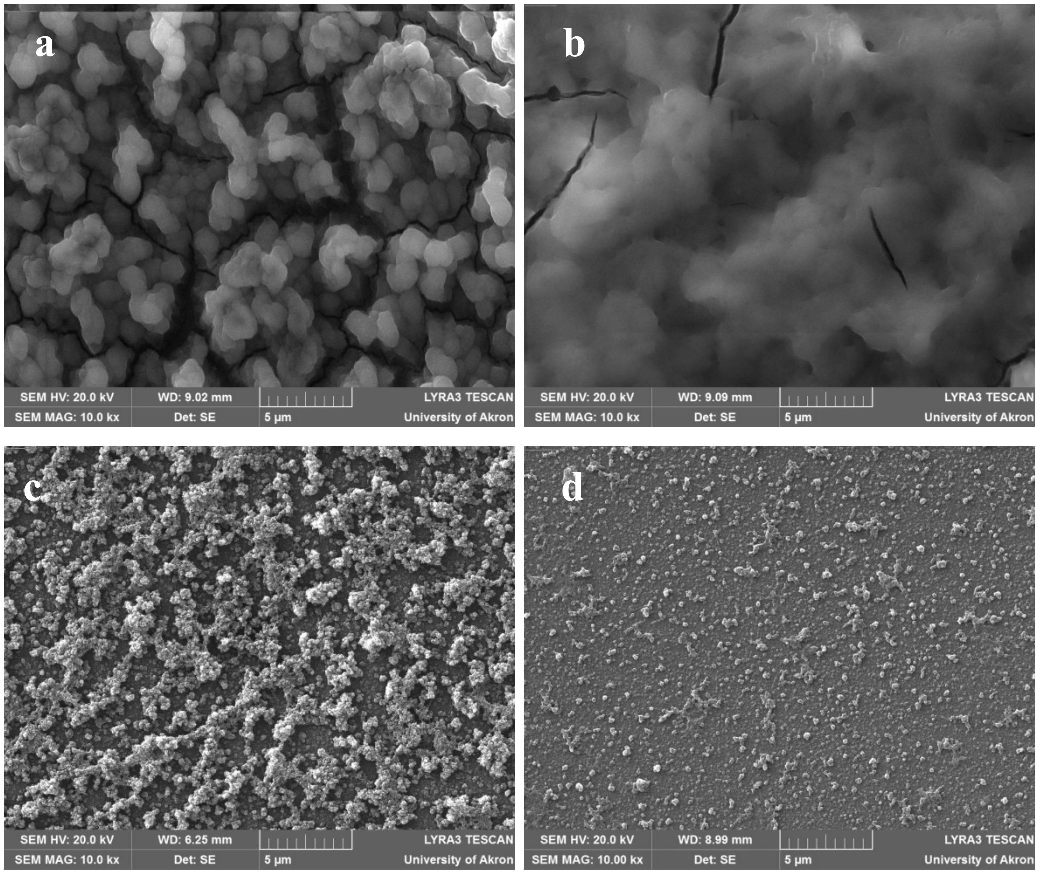

Figure s2**.** Scanning electron microscope images of (a) PSBEDOT, (b) PSBEDOT-GOx, (c) PEDOT, and (d) PEDOT-GOx surfaces electro-polymerized on the ITO surface through galvanostatic method with 1.59 mA/cm^2 for 30 s.

1. B. Cao, C. J. Lee, Z. Zeng, F. Cheng, F. Xu, H. Cong and G. Cheng, *Chem Sci,* 2016, **7**, 1976-1981.
